# Evaluation of the tubular penetration of two different types of nanoparticle root canal sealers over apically separated files: a scanning electron microscopic study (in vitro study)

**DOI:** 10.1186/s12903-025-06263-0

**Published:** 2025-06-03

**Authors:** Alaa H. Nagdi, Nayera A. Mokhless, Mahmoud R. Aboelseoud

**Affiliations:** Division of Endodontics, Conservative Dentistry Department, Faculty of Dentistry, Alexandria University, Champollion St, P. O. Box: 21527, Azarita, Alexandria Egypt

**Keywords:** Apically separated file, Bioactive glass nanoparticles, Bioceramic sealer, Resin sealer, Silver nanoparticles, Tubular sealer penetration

## Abstract

**Background:**

The separation of root canal instruments can significantly impact the quality of canal obturation and the long-term success of endodontic treatment. Combining the benefits of nanoparticles and ultrasonic activation aim to enhance tubular sealer penetration and achieve proper sealing to improve outcome in such complex cases.

**Aim of the study:**

This study aimed to evaluate tubular penetration of two nanoparticles modified root canal sealers compared to conventional sealers using ultrasonic activation over an apically separated file analyzed by scanning electron microscopy (SEM).

**Materials and methods:**

Forty extracted single canaled mandibular premolar teeth were decoronated and prepared using M-Pro (IMD, Shanghai, China) nickel titanium rotary files which were intentionally separated at apical third. Teeth were randomly divided into four equal groups based on the type of sealer used. Group I: Adseal (Rs) (Meta Biomed, Cheongju, Korea) resin-based sealer, group II: Adseal resin-based sealer modified with silver nanoparticles (Rs/Ag), group III: Ceraseal (BC) (Meta Biomed, Cheongju, Korea) bioceramic sealer and group IV: Ceraseal bioceramic sealer modified with bioactive glass nanoparticles (BC/BG). The tested sealers were applied in all root specimens using lentulo-spiral size #25 (Dentsply Maillefer, Ballaigues, Switzerland) except the BC group where the sealer was injected into the root using plastic delivery tips provided by the manufacturer. All sealers were ultrasonically activated then obturated using cold lateral compaction technique. All samples were sectioned longitudinally in a buccolingual direction and analyzed for tubular sealer penetration using SEM at the area of separated file.

**Results:**

The maximum mean tubular sealer penetration depth, measured in microns, was evident in the BC sealer group (97.00 ± 14.78) followed by the Rs/Ag group (94.12 ± 15.89) with no significant difference between them (*p* = 1.00). However, a significant difference (*p* < 0.05) was found when comparing these groups to the Rs group (73.00 ± 7.53) and the BC/BG group (69.78 ± 15.19). No significant difference (*p* = 1.00) was observed between the Rs and BC/BG groups.

**Conclusions:**

Under the parameters of this in vitro study, conventional bioceramic sealer and resin-based sealer modified with silver nanoparticles exhibit more tubular penetration depth over apically separated file compared to conventional resin-based sealer and bioceramic sealer modified with bioactive glass nanoparticles.

## Background

The primary goal of endodontic treatment is to clean, shape, and seal the root canal system, effectively eliminating infection and promoting the healing of periapical tissues [1]. The fracture of endodontic files can occur for various reasons and creates challenges to effective root canal treatment [2]. Leaving the separated file, particularly in the apical third, may does not compromise the outcome mainly if a proper seal is achieved [3–5].

Sealers play a critical role in endodontic treatment, as effective tubular penetration isolates residual bacteria, thereby minimizing the risk of reinfection. The depth and quality of sealer penetration directly affect the success rate of the treatment [6]. Resin-based sealers have been widely used due to their favorable physicochemical characteristics, high bond strength, and minimal polymerization shrinkage [6, 7]. Bioceramic sealers, on the other hand, have gained popularity for their biocompatibility, bioactivity, and ability to chemically bond with the dentinal tubules by forming a hydroxyapatite layer at the interface [8].

Nanomaterials have unique physicochemical properties due to their nanoscale sizes and high surface area to volume ratio [9, 10]. Nanoparticles can penetrate deeper into the dentinal tubules and root canal irregularities, providing a better seal and this is critical for isolating any residual bacteria, thereby reducing the risk of reinfection [11, 12]. Silver nanoparticles have strong antibacterial properties while also enhancing the sealer’s penetration into fine dentinal tubules. This improves interaction with bacteria that often persist within the complex root canal system, thereby enhancing the success of endodontic treatment [12]. Furthermore, bioactive glass nanoparticles can positively improve sealing ability. The release of calcium, silicon and other ions from bioactive glass aids in forming hydroxyapatite within the dentinal tubules, improving the sealing and reducing the risk of reinfection [13].

Recent advancements had focused not only on improving the base materials of sealers but also on enhancing their properties through the incorporation of nanoparticles and activation techniques such as ultrasonic activation had been shown to significantly enhance sealer’s flowability and penetrability into dentinal tubules [14].

Therefore, this in vitro study aimed to evaluate the tubular penetration depth of nanoparticle-modified root canal sealers compared to conventional sealers, utilizing ultrasonic activation over an apically separated file.

The null hypothesis was that there would be no significant difference in dentinal tubule penetration depth between nanoparticle-modified sealers and conventional sealers over apically separated file.

## Materials and methods

### Sample size calculation

This study was accepted by the ethical committee at the Faculty of Dentistry, Alexandria University (serial #0622-02/2023). The minimal sample size was calculated based on a previous study [12], the effect size was calculated as 0.715. The G*Power version 3.1.9.2 [15] was used to calculate the power and it was determined that a total of 40 single canaled mandibular premolar teeth were needed for the study.

### Samples preparation

**Fig. 1:**
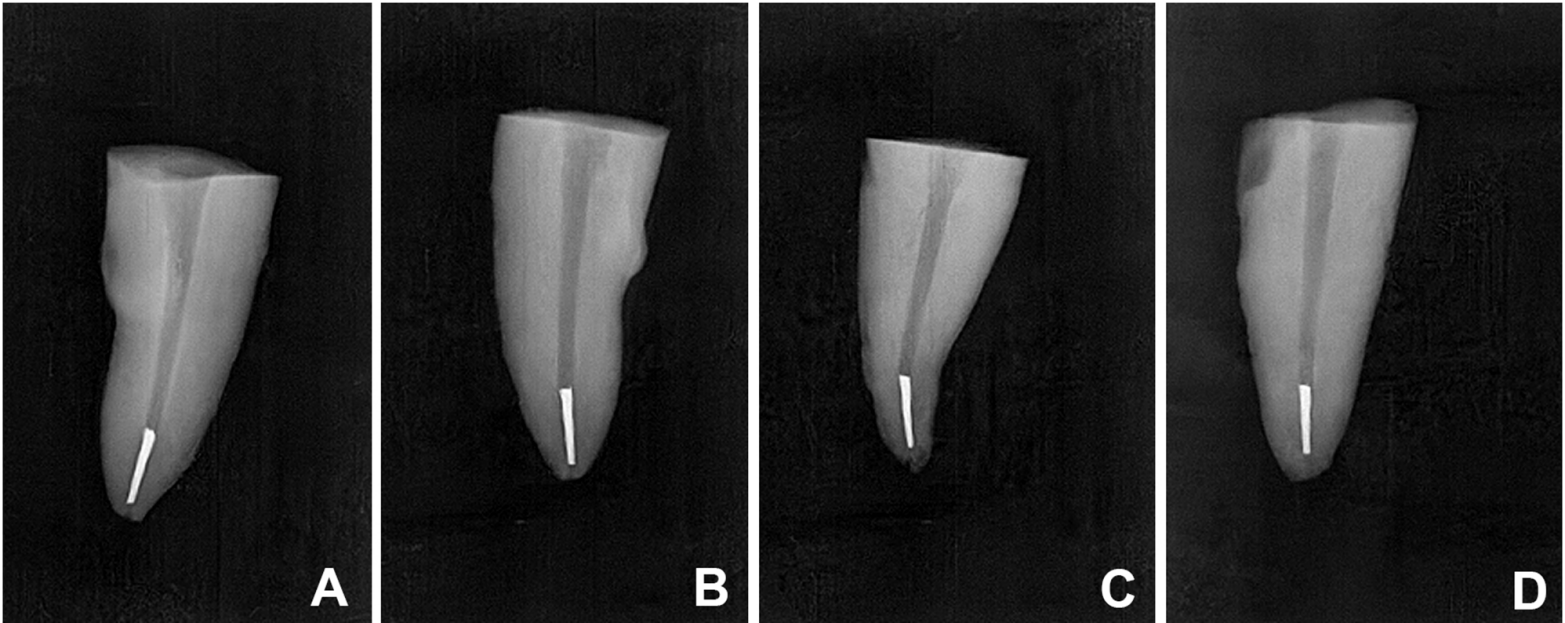
Radiographic images of approximately 3 mm apically file fragment

Forty mature single-rooted mandibular premolar human teeth were collected from Oral and Maxillofacial Surgery Department at Faculty of Dentistry, Alexandria University, Egypt. All subjects gave their informed consent for the inclusion of their extracted teeth. Magnification and radiographs were used respectively to assess the presence of cracks and fractures induced during tooth extraction, presence of any anatomical canal variations and to confirm presence of a single patent canal (Vertucci type I) with moderate curvature ranged from 10 to 25 degrees according to Schneider’s classification [16]. Teeth with root resorption, open apex, root fractures or calcification were excluded. All samples used in this study were evaluated using mesiodistal and buccolingual radiographs to minimize variability in canal morphology. Teeth were cleaned from gross debris or calculus then were preserved in saline solution [12]. All samples were decoronated with a low speed cutting disc (Diatech, Heerbrugg, Switzerland) with water spray cooling to a standardized length of 16 mm. Working length was determined by placing a manual #10 K-file (Mani, Tochigi, Japan) into the root canal until visualized under a magnification at the apical foramen then 1 mm was subtracted from this length [12, 17]. M-Pro (IMD, shanghai, China) rotary file system was used following the order (#18/0.04, #20/0.04) according to manufacturer’s instructions driven by X-Smart Plus (Dentsply Maillefer, Ballaigues, Switzerland) endodontic motor. Irrigation between each file was performed with 3 ml of 5% sodium hypochlorite (NaOCl) (JK dental vision, Egypt) using a 27-gauge side vent needle (PPH CERKAMED, Stalowa Wola, Poland). A diamond disc was used to create a notch 3 mm from the tip of the #25/0.06 file. When the notched file was inserted into the root canal, the engagement was checked with resistant feeling. If file was found to be loose, this sample was excluded and a new sample was prepared following the respective protocol. Controlled rotation pressure was applied to the notched files until separation occurred. If the notched file began to rotate within the canal and the fracture time exceeded 1–2 s, the sample was also excluded [18]. Periapical radiographs were taken to confirm the location of the separated files for all samples in all experimental groups (Fig. 1). Final irrigation was performed with 3 ml of 17% ethylenediaminetetraacetic acid (EDTA) (JK dental vision, Egypt) followed by 3 ml of 5% NaOCl and final rinse with 5 ml distilled water then dried with paper points (Meta Biomed Co, Cheongju, Korea).

### Preparation of nanoparticles and nanoparticles-modified sealers (Table 1)

#### Preparation of silver nanoparticles (AgNPs)

**Fig. 2:**
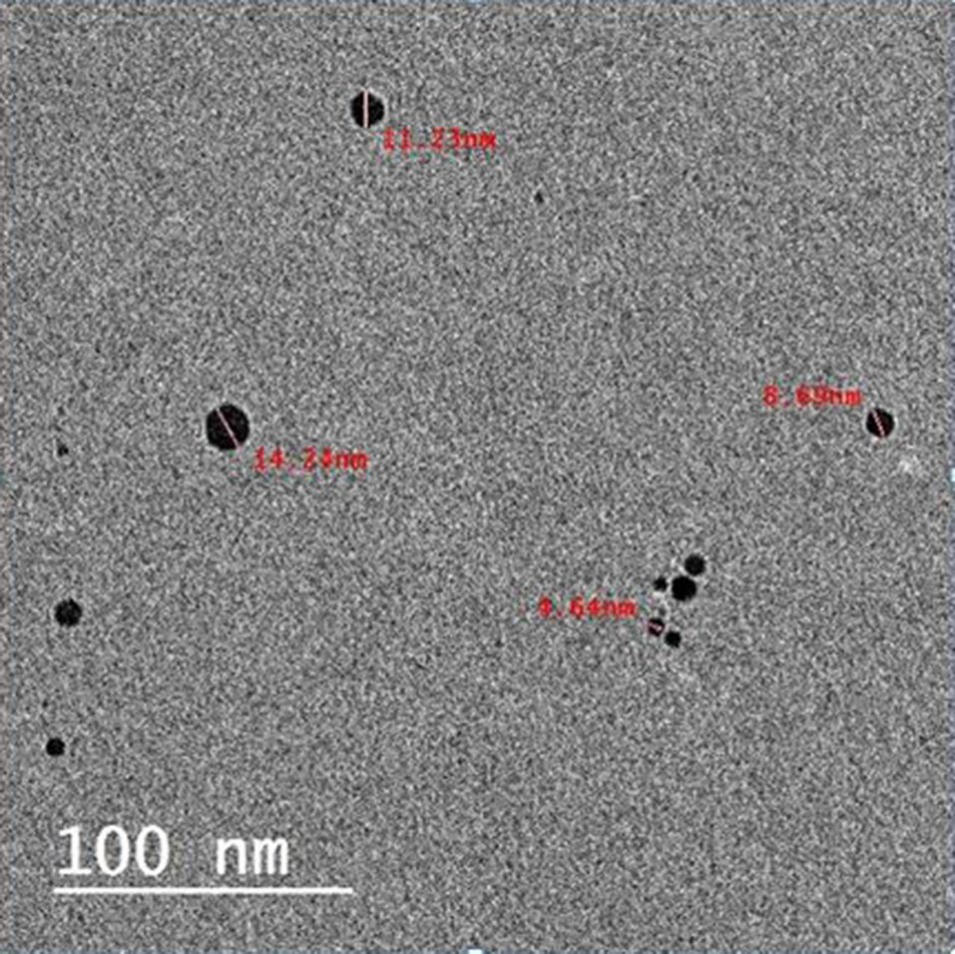
TEM image of AgNPs

Silver nanoparticles were prepared by chemical reduction method. A solution of AgNO3 was used as Ag + 1 ions precursor. Polyvinylpyrrolidone (PVP) was used as stabilizing agent and borohydrate as mild reducing agent. The color of the solution slowly turned into grayish yellow, indicating the reduction of the Ag + 1 ions to Ag nanoparticles [19]. The characterization of AgNPs was performed through optical and morphological analyses. UV-Vis absorption spectra were recorded using an Ocean Optics USB2000 + VIS-NIR fiber optics spectrophotometer. Transmission electron microscopy (TEM) analysis revealed that AgNPs exhibited a deep brown color and a semi-powder form, with an average size of 10 ± 3 nm and a spherical morphology. TEM imaging was performed using a JEOL JEM-2100 high-resolution transmission electron microscope at an accelerating voltage of 200 kV. (Fig. 2)

#### Preparation of bioactive glass nanoparticles (BGNPs)

**Fig. 3:**
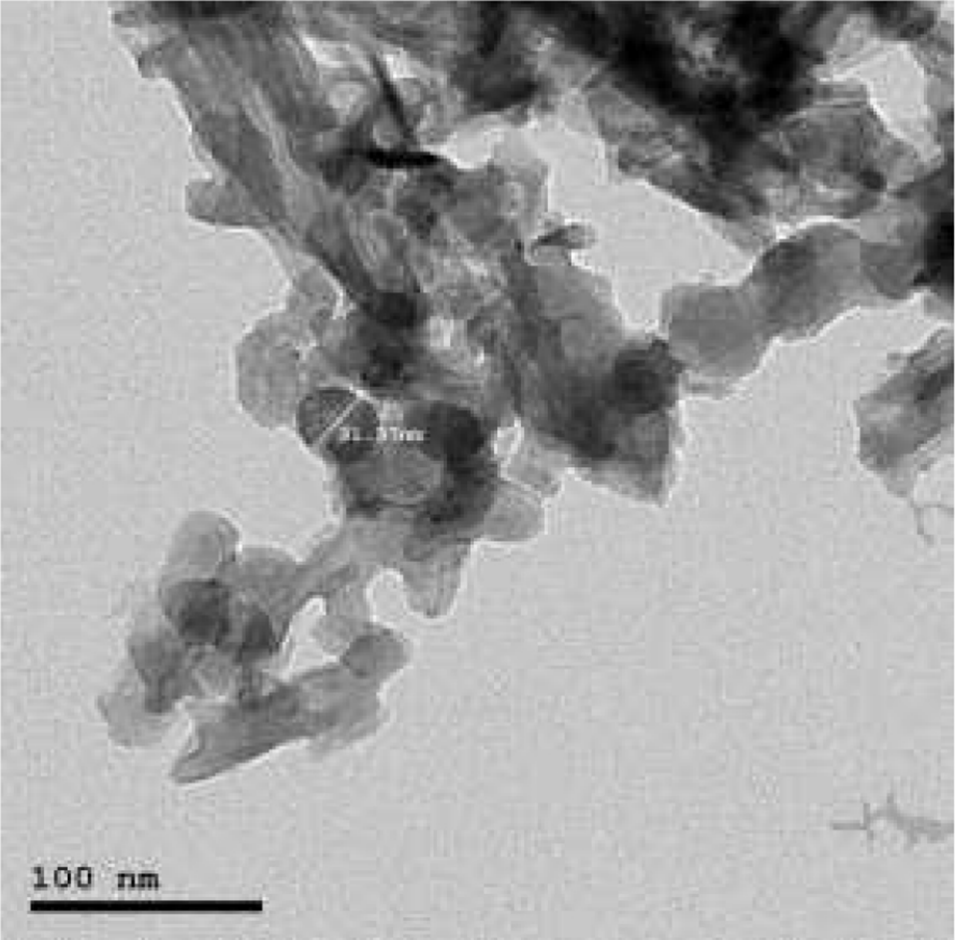
TEM image of BGNPs

The production of bioactive glass nanoparticles was carried out through sol-gel method. It is a wet-chemical technique that can be used for the fabrication of both glassy and ceramic materials [20]. The precursors used for BGNPs synthesis included tetraethyl orthosilicate, calcium nitrate tetrahydrate, Pluronic P123, and ammonia solution were catalyzed and dissolved in the solvent to form a sol. The sol gradually became a gel-like diphasic system containing both a liquid and a solid phase. The morphology ranged from dispersed particles to continuous polymer-like networks, which was mainly dependent on the pH and solvent. Generally, the sol–gel processes included hydrolysis, polycondensation, drying, and stabilization. To strengthen the gelation, aging network, an aging process was necessary. Removal of the remaining liquid solvent phase required a drying process. Finally, a thermal treatment (stabilization) carried out to enhance the mechanical properties and to improve the structural stability [21]. The characterization of BGNPs was performed through morphological analysis. TEM analysis showed that BGNPs appeared as a white powder with an average size of 50 ± 20 nm and a spherical morphology. TEM imaging was performed using a JEOL JEM-2100 high-resolution transmission electron microscope at an accelerating voltage of 200 kV. (Fig. 3)

#### Preparation of sealers modified with nanoparticles

Resin-based sealer was dispensed onto a glass slab and manually mixed using a plastic spatula for approximately 10–15 s at room temperature, following the manufacturer’s instructions to ensure a homogeneous consistency, then AgNPs were incorporated immediately after the initial mixing phase and fully integrated within the same period to maintain consistency. In the present study, 0.15% (wt/wt) AgNPs were incorporated into the resin-based sealer, following the findings of Seung J. et al. (2018). Their results demonstrated that the addition of 0.15% AgNPs did not result in significant changes to setting time, flow, solubility, or dimensional stability, thereby supporting its application without compromising the physical integrity of the sealer [22].

The bioceramic sealer was dispensed onto a glass slab and manually mixed with a plastic spatula with BGNPs until a homogeneous mixture was achieved. BGNPs were incorporated at a concentration of 1% (wt/wt), based on the findings of Jung et al. (2022), whose study demonstrated that this concentration provided a favorable balance between enhanced bioactivity and maintained physicochemical properties within acceptable limits [13]. To preserve the sealer’s effectiveness and avoid exceeding its mixing and working time, all sealers across all the experimental groups were prepared and applied in pairs (two samples at a time), minimizing delays between mixing and obturation.

**Table 1:** Materials used

Material	Commercial product name	Manufacturer	Composition	Lot No.
Resin-based sealer	Adseal	Meta Biomed, Cheongju, Korea	Base Paste: epoxy oligomer resin, ethylene glycol salicylate, and bismuth subcarbonate. Catalyst Paste: Comprises polybutanediol aminobenzoate, calcium phosphate, and bismuth subcarbonate.	ADP2410071
Bioceramic sealer	Ceraseal	Meta Biomed, Cheongju, Korea	Zirconium Dioxide (ZrO₂): 45–50%, Tricalcium Silicate (Ca₃SiO₅): 20–30%, Dicalcium Silicate (Ca₂SiO₄): 1–10%, Tricalcium Aluminate (Ca₃Al₂O₆): 1–10%, Thickening Agents: trace amounts	CSL2410101
Silver nanoparticles	Silver nanoparticles	Nano Tech Co., Cairo, Egypt	95% Polyvinylpyrrolidone (PVP) 5% Silver (AgNPs)	
Bioactive glass nanoparticles	Bioactive glass nanoparticles	Nano Tech Co., Cairo, Egypt	15 mol% Calcium oxide 85 mol% Silicon dioxide	

### Chemical characterization of sealers modified with nanoparticles

The nanoparticle-modified sealers, prepared according to the selected nanoparticle concentrations, were allowed to set completely under controlled conditions at 23 ± 1 °C and 100% humidity for 72 h, simulating clinical setting conditions. After setting, the specimens were manually ground into fine powders using a mortar and pestle and subsequently subjected to X-ray diffraction (XRD) and Fourier-transform infrared spectroscopy (FTIR) analyses. For XRD, phase analysis was conducted to identify crystalline phases, determine crystal structures, and investigate material properties at the atomic level [23]. FTIR analysis was performed using an infrared spectrophotometer to identify chemical bonds, functional groups, and molecular structures within the experimental modified sealers [23].

### Obturation

All specimens were given numbers and randomly divided into four equal groups (*n* = 10). Using permuted block technique, where samples were assigned in blocks of 4 [24].

Study group I: Resin-based sealer (Rs).

Study group II: Resin-based sealer modified with silver nanoparticles (Rs/Ag).

Study group III: Bioceramic sealer (BC).

Study group IV: Bioceramic sealer modified with bioactive glass nanoparticles (BC/BG).

**Fig. 4:**
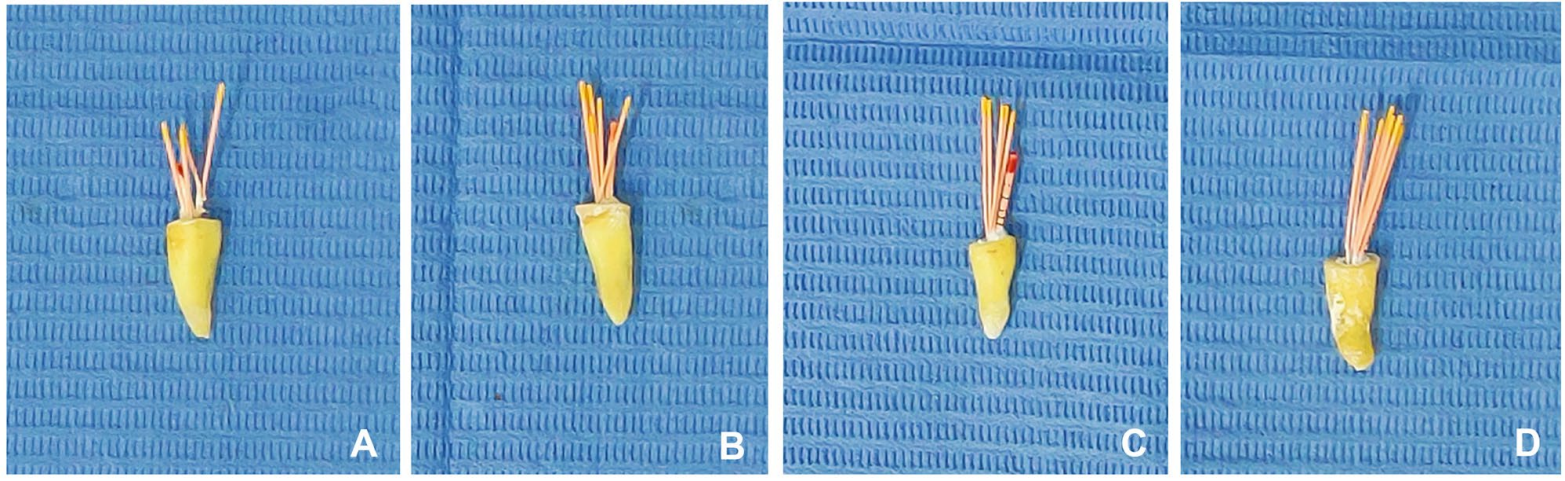
Cold lateral obturation for each group

**Fig. 5:**
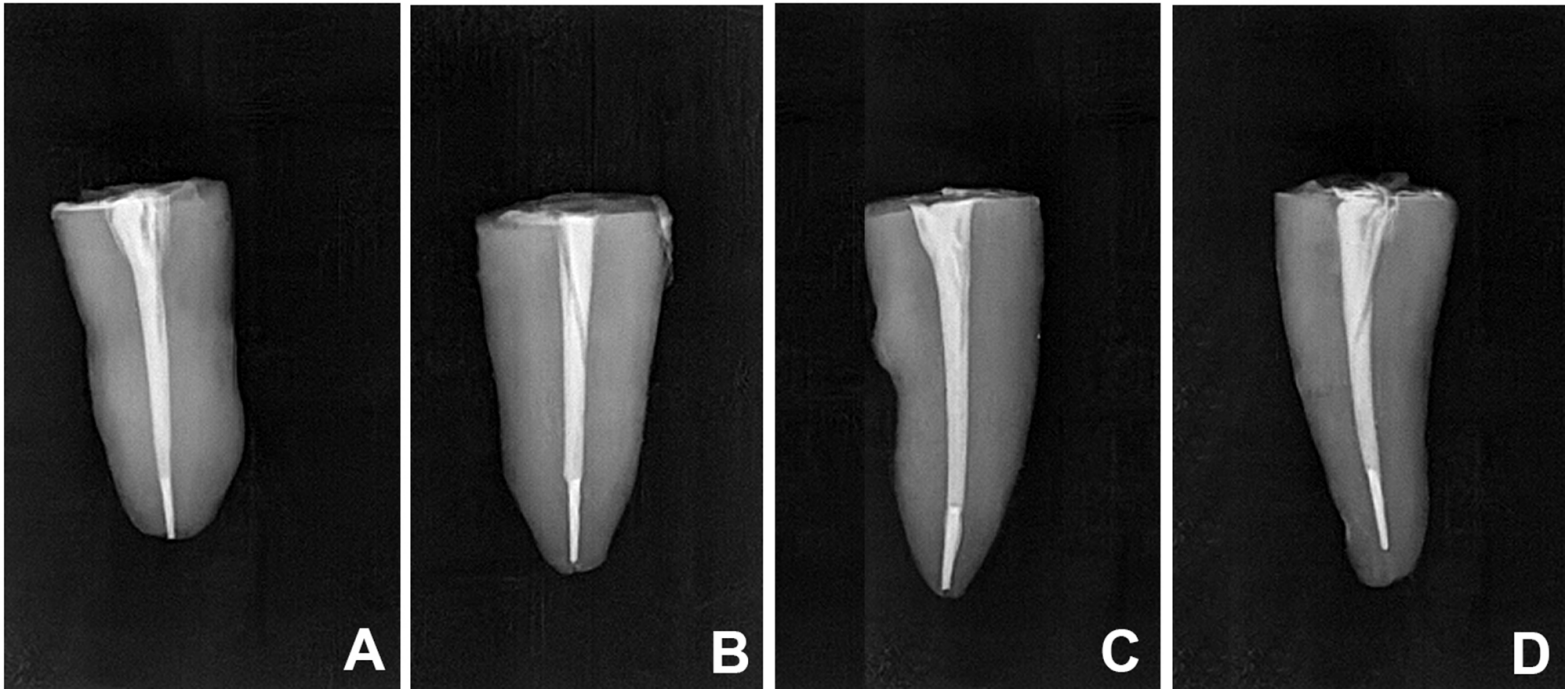
Radiographic images after obturation for each group

All canals were coated with assigned sealers using a lentulo-spiral size #25 according to each group except the BC group where the sealer was injected into the root using plastic delivery tips. Then, ultrasonic activation was carried out using E4 non-cutting tip (Woodpecker, Guilin, China), mounted on UDS-A (Woodpecker, Guilin, China) ultrasonic device set to the endo mode with a power setting of 5. As the ultrasonic device oscillates in a linear plane, the tip was activated for 20 s in the buccolingual direction and for an additional 20 s in the mesiodistal direction of the root canal [25]. Gutta-percha (Meta Biomed, Cheongju, Korea) that matched the size of the separated file (#25/0.06) was trimmed 3 mm from the tip with a scalpel, then coated with sealer and applied over the separated file and obturation was completed by cold lateral compaction technique using #25 finger spreader (Mani, Tochigi, Japan) and #20 accessory cones (Meta Biomed, Cheongju, Korea) (Fig. 4). Excess gutta-percha points were seared with a hot instrument and condensed vertically with a plugger. The root canal orifices were sealed with temporary filling material (BMS, Capannoli, Italy). All obturation procedures in all groups were performed by a single operator. Buccolingual and mesiodistal periapical radiographs were taken to evaluate the quality of obturation (Fig. 5). All specimens were stored at 37 °C and 100% humidity for 1 week in a humidifier to allow the sealers to set [12].

### Samples preparation and analysis using scanning electron microscope (SEM)

**Fig. 6:**
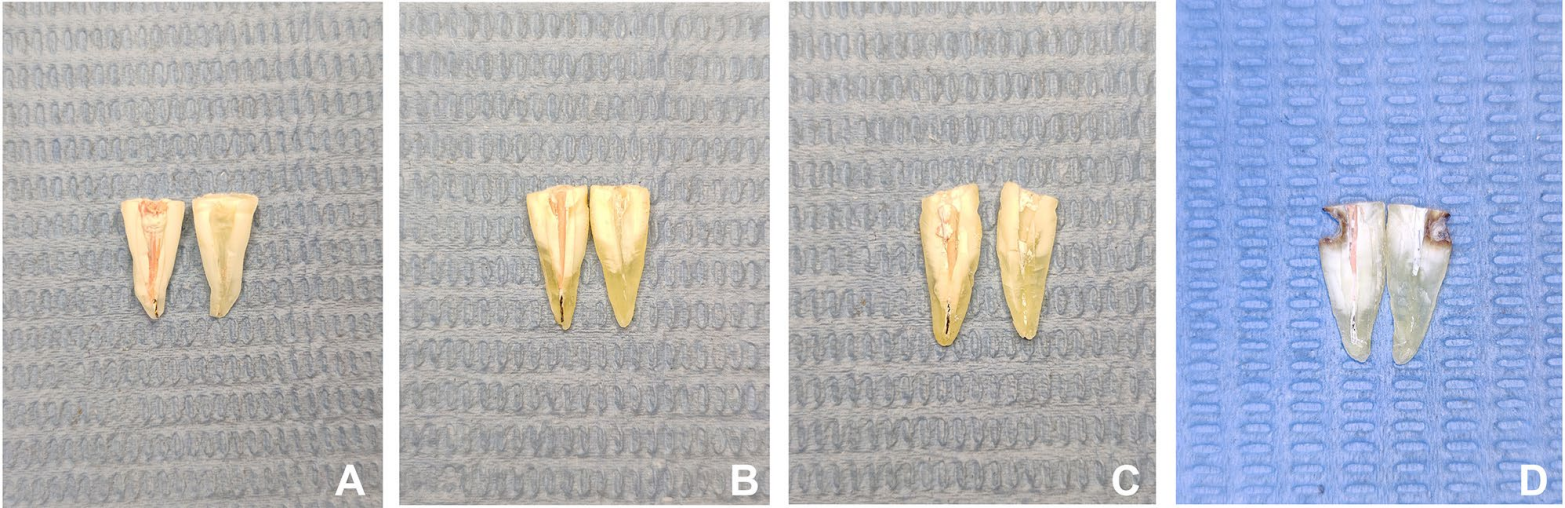
Sectioning samples after obturation for each group

Specimens were longitudinally sectioned in a buccolingual direction by creating a thin slot along the buccal and lingual aspects of the root using a diamond disk at low speed under continuous water cooling, followed by splitting with a chisel and mallet [12] (Fig. 6). Sealer penetration depth was then analyzed using SEM, starting from the dentinal tubules near the coronal part of the fractured file and progressing apically. The canal wall was used as a reference point until sealer penetration was no longer observed. Sealer penetration depth was measured using ImageJ software (NIH, Bethesda, MD, USA). SEM images were first calibrated using the embedded scale bar to convert pixel values to micrometers, then linear measurements were performed by drawing lines from the canal wall to the maximum and minimum points of visible sealer penetration into the dentinal tubules for each experimental sample [26]. The mean tubular sealer penetration depth was calculated specifically in the region of the apically separated file. To assess measurement consistency, intra-observer reliability was evaluated by having the same examiner re-analyze 20% of the images after a two-week interval. Inter-observer reliability was not assessed in the current study, as all measurements were performed by a single trained operator. Reliability was measured using the Intraclass Correlation Coefficient (ICC) with a two-way mixed-effects model and absolute agreement. ICC values greater than 0.90 were interpreted as indicating excellent agreement [27].

### Statistical analysis

Normality of the continuous data were tested using Kolmogorov-Smirnov and Shapiro-Wilk tests. Continuous data were described by the mean, standard deviation (SD), minimum, maximum, median and interquartile range (IQR). The difference in the tubular sealer penetration depth was assessed using the One-way ANOVA test followed by the Bonferroni post hoc test. Significance level was set a *p* < 0.05. Data were analysed using SPSS version 23.0 for Windows (SPSS Inc., Chicago, USA).

## Results

### X-ray diffraction (XRD)

#### Resin-based sealer modified with silver nanoparticles (Rs/Ag) (Fig. 7a)

**Fig. 7:**
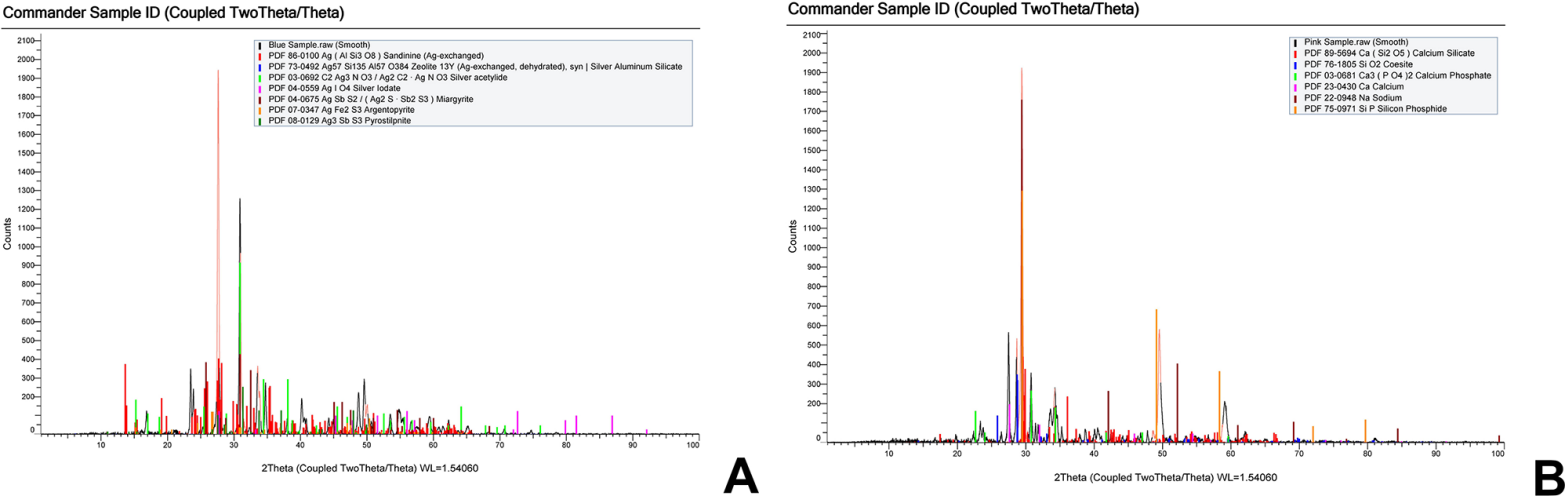
XRD results (**a**) Rs/Ag group, (**b**) BC/BG group

Pure silver nanoparticles peaks were found at 38.0 °2θ, 44.3 °2θ, and 65.0 °2θ. These peaks were much less prominent and weaker than the peaks where silver was combined with other elements that are originally present in the sealer. In the XRD diffractogram, silver was bound to silicon (26.0 °2θ, 28.1 °2θ, 31.3 °2θ, 35.3 °2θ, 40.2 °2θ), nitrogen (17.0 °2θ, 30.9 °2θ, 34.4 °2θ, 38.0 °2θ, 53.5 °2θ), and iron (30.9 °2θ, 34.0 °2θ, 50.3 °2θ). Radio-opacifiers such as bismuth oxide could be found at 25.1 °2θ, 27.3 °2θ, 34.8 °2θ, 38.0 °2θ, 44.3 °2θ, and 55.5 °2θ. Silica filler peaks were present at 25.8 °2θ, 27.3 °2θ, 30.9 °2θ, 40.2 °2θ, 45.0 °2θ, and 51.8 °2θ [28–31].

#### Bioceramic sealer modified with bioactive glass nanoparticles (BC/BG) (Fig. 7b)

The most dominant and overlapping peaks in the XRD analysis belonged to calcium silicate, calcium carbonate, and calcium silicate hydrate peaks which were found at 23.5 °2θ, 29.5 °2θ, 33.4 °2θ and 36.0 °2θ. At 28.7 °2θ a prominent peak of silicon oxide could be found. Calcium phosphate was observed at 22.6 °2θ, 30.8 °2θ, and 34.3 °2θ. Silicon phosphate could be observed with a sharp intense peak at 29.4 °2θ along with two peaks at 49.1 °2θ and 58.3 °2θ. Sodium peaks belonging to bioactive glass could be seen at 29.4 °2θ, 52.1 °2θ, and 61.0 °2θ [32–34].

### Fourier transform infra-red spectroscopy (FTIR)

#### Resin-based sealer modified with silver nanoparticles (Rs/Ag) (Fig. 8a)

**Fig. 8:**
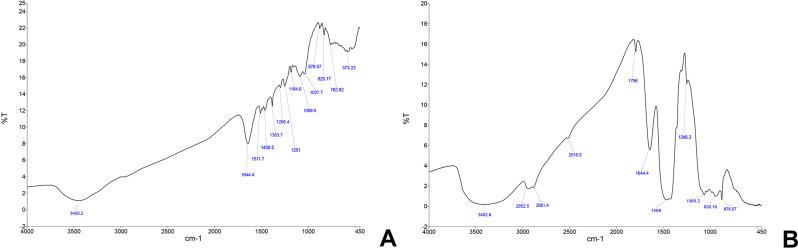
FTIR results (**a**) Rs/Ag group, (**b**) BC/BG group

Stretching of OH and NH groups were found at 3402.8 cm^− 1^. An asymmetric stretching CH band was found at 2955.0 cm^− 1^, while the symmetric stretch was found at 2866.0 cm^− 1^. The CH bending vibrations were seen at 1459.5 cm^− 1^, 1251.0 cm^− 1^, and 736.5 cm^− 1^. The C = C bonds of the resin were represented at 1644.4 cm^− 1^. The peaks at 1511.7 cm^−^1, 829.1 cm^− 1^ and 762.8 cm^− 1^ indicated the presence of aromatic groups and rings. The band of 1488.4 cm^− 1^ represented the aromatic nitro compounds and the ones at 1383.7 cm^− 1^and 829.1 cm^− 1^ were attributed to nitrate ions. C–N stretching was represented by the bands 1383.7 cm-1 and 1037.7 cm-1. Asymmetric stretching bands of bridging silicon Si-O-Si were found at 1184.6 cm^− 1^, while the symmetric ones were found at 803.7 cm^− 1^ and their bending vibrations were found at 453.2 cm^− 1^. The bands at 534.1 cm^− 1^ and 504.2 cm^− 1^ were characteristic for Ag-O [28–31].

#### Bioceramic sealer modified with bioactive glass nanoparticles (BC/BG) (Fig. 8b)

A broad OH stretching band was detected at 3402.8 cm⁻¹, resulting from the hydroxyl group of bioactive glass and the sealer hydration. An asymmetric stretching CH band was found at 2952.5 cm⁻¹, while the symmetric stretch was observed at 2881.4 cm⁻¹. The CH bending vibrations were seen at 1469.0 cm⁻¹ and 1246.3 cm⁻¹. The C-O stretching appeared at 2516.5 cm⁻¹ and 1796.0 cm⁻¹, indicating the presence of calcium carbonate. The peak at 1644.4 cm⁻¹ indicated the presence of aromatic groups and C = C bonds. The same band, along with 3402.8 cm⁻¹, indicated the presence of calcium silicate hydrate bonds. The stretching found at 1422.1 cm⁻¹ was attributed to the presence of CaCO₃ or carbonated apatite. The vibrations at 1356.6 cm⁻¹ represented carboxylic groups, which might have resulted from the interaction of bioactive glass with calcium carbonate. Stretching bands of bridging silicon Si-O-Si were found at 1032.3 cm⁻¹, and their bending vibrations were observed at 456.2 cm⁻¹. At 979.4 cm⁻¹ and 843.6 cm⁻¹, Si-O was detected, which could be attributed to calcium silicate. The bands at 717.8 cm⁻¹ and 578.7 cm⁻¹ described the calcium sodium silicate phase. The phosphate group asymmetric bending vibrations were detected at 618.5 cm⁻¹ and 585.3 cm⁻¹, which showed strong intensity evident by the low percentage transmission of this band due to the presence of such a group in both the sealer and the bioactive glass, as well as their strong attachment to calcium [32–34].

### Sealer penetration into dentinal tubules (Tables 2 and 3)

**Fig. 9:**
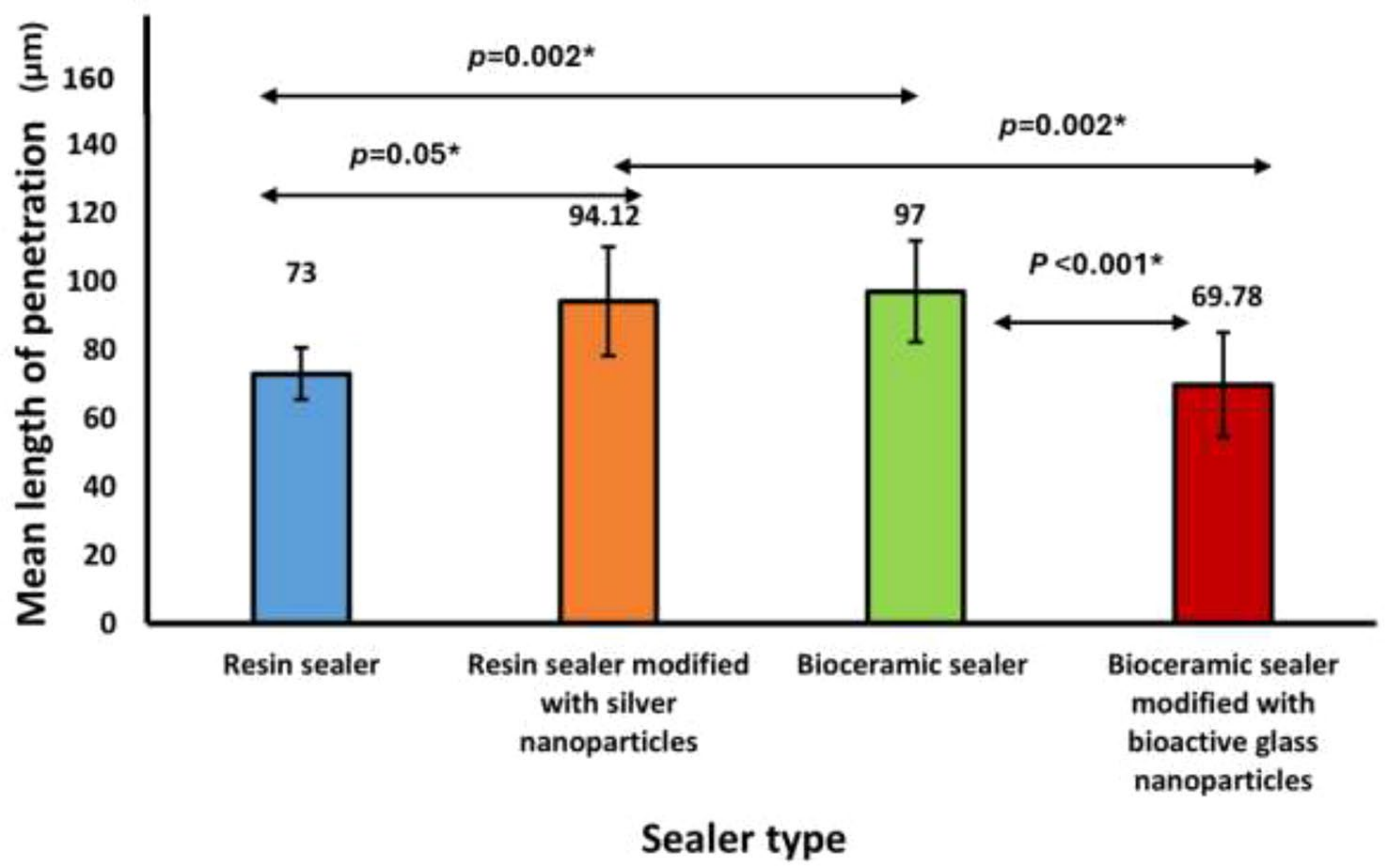
Difference in mean tubular sealer penetration between the tested sealers

**Fig. 10:**
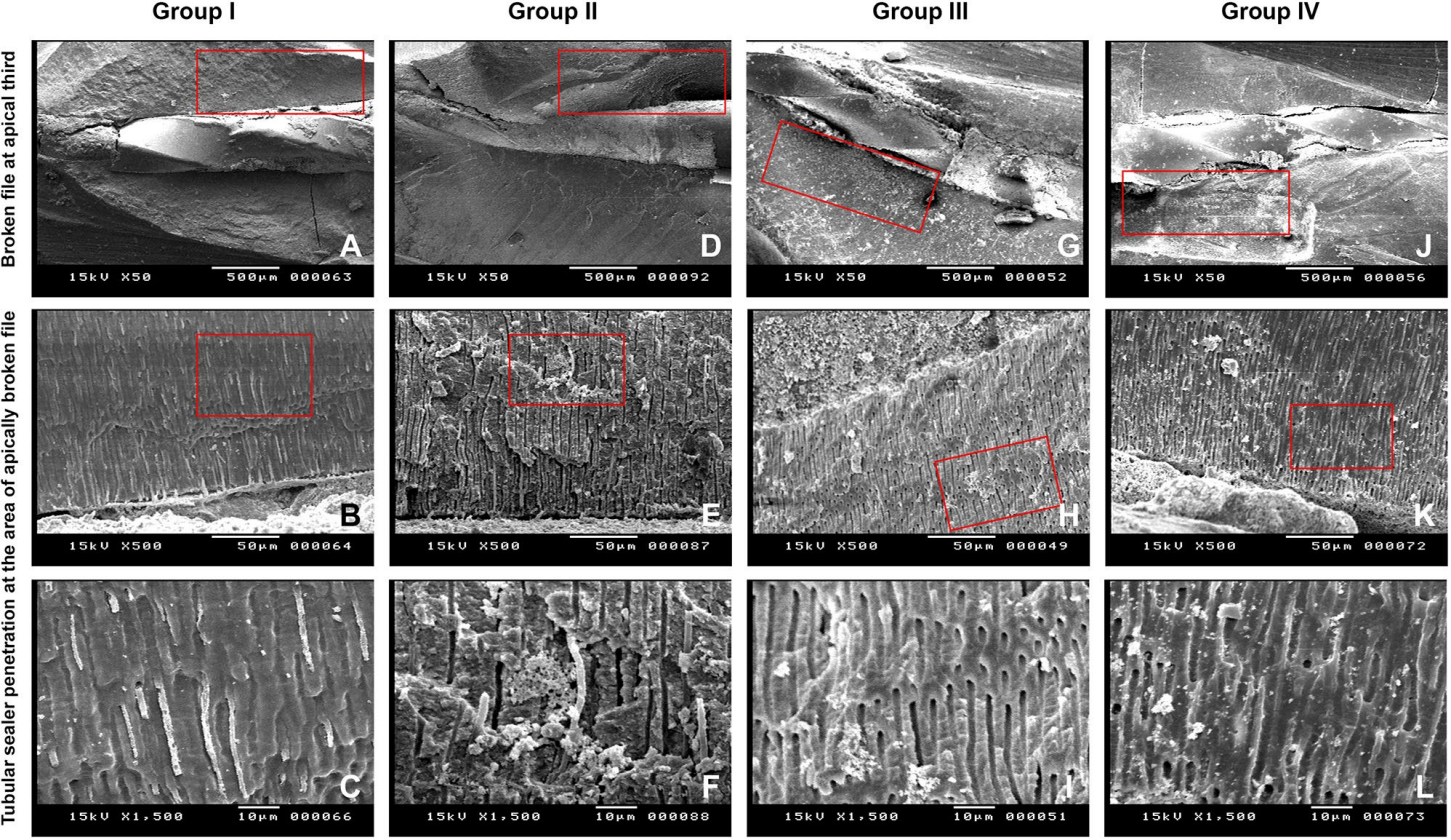
Scanning Electron Microscopy (SEM) images illustrating the fractured file fragment at the apical third and tubular sealer penetration across different experimental groups. (**A**, **D**, **G**, **J**) show the apically separated file fragment within the root canal at X50 magnification. (**B**, **E**, **H**, **K**) highlight tubular sealer penetration at the fractured file area at X500 magnification. (**C**, **F**, **I**, **L**) present high-magnification images at X1500 corresponding to the red-boxed regions, revealing the depth of tubular sealer penetration in each group

The maximum mean tubular sealer penetration depth, measured in microns was seen in the BC sealer group (97.00 ± 14.78) followed by the Rs/Ag group (94.12 ± 15.89) with no significant difference between them (*p* = 1.00). However, a significant difference (*p* < 0.05) was found when comparing these groups to the Rs group (73.00 ± 7.53) and the BC/BG group (69.78 ± 15.19). No significant difference (*p* = 1.00) was observed between the Rs and BC/BG groups. (Figures 9 and 10)

**Table 2:** Difference in tubular sealer penetration depth between the tested sealers in micrometres (*N* = 40)

Sealer type	Penetration depth				*p* value
	Minimum	Maximum	Median (IQR)	Mean ± SD	
Resin sealer (Rs)	59.53	82.79	71.43 (69.14, 81.84)	73.00 ± 7.53	< 0.001*
Resin sealer modified with silver nanoparticles (Rs/Ag)	72.40	120.40	90.80 (81.85, 109.65)	94.12 ± 15.89	
Bioceramic sealer (BC)	75.80	119.60	93.60 (84.20, 111.70)	97.00 ± 14.78	
Bioceramic sealer modified with bioactive glass nanoparticles (BC/BG)	54.60	100.40	66.90 (56.10, 77.85)	69.78 ± 15.19	

*IQR* interquartile range, *SD* Standard deviation

One-way ANOVA test

*Statistically significant at *p* < 0.05

**Table 3:** Post hoc test for the difference in sealer penetration between the types of sealers in micrometres (*N* = 40)

Sealer type	Compared to	*p* value
Resin sealer (Rs)	Resin sealer modified with silver nanoparticles	0.01*
	Bioceramic sealer	0.002*
	Bioceramic sealer modified with Bioactive glass nanoparticles	1.00
Resin sealer modified with silver nanoparticles (Rs/Ag)	Bioceramic sealer	1.00
	Bioceramic sealer modified with Bioactive glass nanoparticles	0.002*
Bioceramic sealer (BC)	Bioceramic sealer modified with Bioactive glass nanoparticles	< 0.001*

Bonferroni post hoc test

*Statistically significant at *p* < 0.05

## Discussion

The persistence of microbial infection within the root canal system is a major factor contributing to the failure of endodontic treatment. The primary goal of endodontic therapy is to eradicate these microorganisms and prevent reinfection [35]. However, achieving complete cleaning and disinfection becomes particularly challenging in cases involving separated endodontic files, which present a significant obstacle and have a reported clinical incidence of 2.27% [36]. File separation is associated with complex root canal anatomy, excessive torque, and cyclic fatigue of the instrument [36–38]. In many cases, clinicians choose to leave the separated fragment, especially if located in the apical third to avoid complications like perforation or excessive removal of dentin [2, 3]. Studies showed that the prognosis of root canal treatment involving retained separated instruments was influenced by several factors, including the degree of canal disinfection prior to instrument fracture, the location of the fracture, the presence of preoperative periapical lesions, and the ability to achieve a proper seal above the separated instrument, with evidence suggested that fractures occurring after canal disinfection and in the absence of apical periodontitis were associated with a favorable prognosis, emphasizing the importance of adequate cleaning before the fracture and achieving an effective apical seal after obturation [4, 5, 16].

As demonstrated by several studies, tubular sealer penetration plays a crucial role in enhancing sealing ability by increasing the contact area between the filling material and dentin, which may contribute to the overall success of endodontic treatment [6, 39, 40]. Regarding these considerations, we aimed to enhance deeper tubular penetration by adding specific types of nanoparticles to different sealers with ultrasonic activation to sealers and cold lateral compaction technique for obturation over apically separated file that may result in enhancing the sealing efficacy in such challenging conditions.

In this study, teeth extracted for periodontal reasons were selected due to their tendency to exhibit narrower pulp spaces. Periodontal disease is often associated with gingival recession and loss of the cementum layer, which can result in the exposure of dentinal tubules and lateral canals to the oral environment which facilitates microbial ingress, potentially eliciting a defensive response from the pulp tissue, such as the deposition of tertiary dentin and the development of calcific metamorphosis. These pathological changes are known to reduce the canal lumen, thereby increasing the complexity of root canal instrumentation and the likelihood of procedural errors such as instrument separation [41, 42].

The M-Pro rotary system was used in this study due to a flaw in the file sequence design which follows the sequence (#18/0.04, #20/0.04, #25/0.06), where the transition from smaller file diameter and tapering to larger diameter and increased taper makes these files more susceptible to fracture. The abrupt increase in size and taper can generate excessive stress on the instrument during canal preparation, increasing the risk of file separation in challenging anatomical conditions.

In the present study, resin-based sealer was selected for its superior sealing and adhesive properties, along with its antibacterial activity, which help minimize bacterial infiltration and promoting an effective seal. resin-based sealers also have excellent flowability and ability to form a thin film layer facilitating deeper penetration into dentinal tubules, thereby enhancing overall sealing efficiency [43]. Furthermore, the sealer’s low solubility ensures the integrity of the root canal obturation, supporting long-term treatment success [44]. Similarly, bioceramic sealer was chosen due to its biocompatibility and bioactivity, which support mineralization, promote hydroxyapatite formation, and enhance the sealing effectiveness. Moreover, its dimensional stability and minimal shrinkage are essential for maintaining a durable seal and preventing gaps that could compromise treatment outcomes [45–48]. Consequently, both resin-based and bioceramic sealers were selected for their reliable long-term performance and their ability to provide effective sealing of root canal systems.

Furthermore, the selection of nanoparticles was guided by biochemical compatibility. Previous studies revealed that silver nanoparticles were most incorporated into resin-based sealers due to their favorable interaction with resin matrices, without adversely affecting their physical properties [22, 49, 50]. Recent research has explored the role of AgNPs in enhancing the effectiveness of root canal treatments, particularly their impact on tubular sealer penetration. AgNPs enhance the flowability of sealers, facilitating deeper penetration into dentinal tubules, which improves adaptation and strengthens the overall sealing efficiency of the root canal system [12, 22, 51, 52]. Similarly, bioactive glass nanoparticles were added to the bioceramic sealer due to their similarity in chemical composition, particularly their calcium silicate base. BGNPs have gained popularity for their bioactivity, which promotes mineralization, thereby increasing the overall sealing effectiveness [13]. To achieve this result, uniform dispersion and effective incorporation between the sealer and the nanoparticles is essential. The results from XRD and FTIR analyses confirmed the integration of the selected nanoparticles within the sealers in each experimental group.

Results from the XRD analysis of (Rs/Ag) revealed the presence of pure AgNPs incorporated into the resin sealer matrix. Peaks correspond to silver-silicon, silver-nitrogen and silver-iron interactions which may suggest enhanced mechanical properties. Moreover, the reduced intensity of pure silver peaks suggested that silver was predominantly distributed in bound forms within the matrix, enhancing the stability and homogeneity of the modified sealer. The FTIR analysis of the same group (Rs/Ag) demonstrated the chemical integration of AgNPs into the sealer. The detection of C-O stretching and carboxylic groups reflected interactions between the nanoparticles and the sealer matrix. The XRD and FTIR findings collectively showed that the incorporation of AgNPs may enhance mechanical properties and sealing ability, suggesting that the modified sealer might be more effective in root canal treatments.

Analysis from the XRD of (BC/BG) demonstrated the successful integration of BGNPs into the bioceramic sealer matrix. Peaks corresponding to calcium silicate hydrate highlighted the potential for dentinal tubule occlusion, thereby supporting effective sealing. The detection of silicon oxide peaks confirmed the incorporation of silica, contributing to the mechanical strength and stability of the sealer. Additional peaks related to sodium and silicon phosphates indicated interactions between the BGNPs and the sealer components. The distribution of these phases emphasized the compatibility between the BGNPs and the bioceramic sealer. Moreover, the FTIR analysis provided further insights into the chemical interactions within the modified sealer. Broad OH stretching bands confirmed the hydration of the bioceramic sealer and the presence of hydroxyl groups from the BGNPs. Peaks associated with C-O stretching and carboxylic group vibrations revealed interactions between the nanoparticles and calcium-based components, which might support the sealing capabilities.

Ultrasonic activation of sealers was done in the present study as it was shown to enhance the tubular sealer penetration and improve their adaptation to dentin through the activation of solid-liquid systems, inducing physicochemical changes [53]. The increased tubular penetration was further explained by the heat generated during activation, which reduced the sealer’s viscosity and promoted greater flowability [25]. Previous studies showed the effect of ultrasonic activation on reduced void formation, enhanced sealer adaptation, and improved tubular penetration through the acoustic microstreaming energy transmitted into the root canal system [14, 54].

Cold lateral compaction was used as the obturation technique across all study groups for standardization, as previous studies have reported that the choice of obturation technique can directly influence tubular sealer penetration [55, 56]. Moreover, it is simple, cost-effective, and remains the most widely used technique in both clinical and laboratory research, which provides an effective seal in root canals with regular anatomy and demonstrates reliable results when used with resin-based and bioceramic sealers [57–59].

The current study utilized scanning electron microscopy (SEM) to evaluate sealer penetration into dentinal tubules, consistent with methodologies employed in several previous investigations [6, 60]. SEM’s high-resolution imaging and magnification capabilities enabled the assessment of sealer penetration across the entire area of interest, particularly around the region of the apically separated file. This technique allowed visualization of sealer within dentinal tubules, even in areas of low tubule density located farther from the canal wall [61].

Based on the results of this in vitro study, the null hypothesis was rejected, as a significant difference was observed in the tubular penetration depth comparing the four tested sealers in the presence of apically separated instrument. To the best of our knowledge, no published studies have specifically investigated the tubular penetration of nanoparticle-modified sealers over apically separated file, making direct comparisons unavailable. Conversely, numerous studies have evaluated the sealing quality of different obturation techniques over apically separated files using various assessment methods [60–62]. However, these studies focused on the impact of the obturation technique on the sealing efficacy rather than the tubular penetration ability of the sealer itself in the region of the separated file.

Our in vitro study revealed that (Rs) group exhibited less penetration depth compared to (Rs/Ag) group. This might be due to the larger particle size, higher viscosity and limited flow characteristics of resin particles [63]. However, the incorporation of AgNPs into resin-basaed sealer significantly improved penetration depth. The results from our study align with the result by Kishen et al. (2008) who reported that the addition of AgNPs to root canal sealer improved its flow properties, likely due to the nanoscale size of silver particles, which enhance flowability and reduce viscosity, thereby facilitating deeper tubular penetration [64]. Similarly, ElKateb et al. (2015) further reinforced this concept, demonstrating that particle size directly influences the flow properties of a sealer and its ability to penetrate into dentinal tubules. Among the various nanoparticles evaluated, silver nanoparticle-modified sealers exhibited the deepest penetration into dentinal tubules [12].

However, these findings contradict those of Kaplan et al. (2003), who concluded that the flow of the sealer was primarily determined by its final consistency and setting reaction rather than particle size [65]. Additionally, Seung J et al. (2018) observed that incorporating AgNPs into resin sealer significantly reduced its flow; however, this reduction remained within the acceptable limits specified by ANSI/ADA standards [22].

Moreover, the results of this in vitro study demonstrated that the (BC) group exhibited greater tubular penetration depth compared to all other groups. This might be due to its smaller particle size, enhanced flowability, and hydrophilic properties, which together facilitate better penetration and adaptation to dentinal tubules, ensuring a more effective seal [66, 67]. Conversely, the addition of BGNPs to bioceramic sealer negatively affected penetration ability compared to conventional bioceramic sealer. These results are consistent with findings by Jung M-K et al. (2022), who reported that the addition of BGNPs to bioceramic sealer reduced its flowability and increased its density and viscosity, potentially explaining the observed decrease in tubular penetration [13]. These results may be attributed to the formation of calcium silicate hydrate resulting from the reaction between the bioceramic sealer and BGNPs. Calcium silicate hydrate fills the gaps between the dentin and sealer while also grows within and occludes the dentinal tubules, which could limits the sealer’s ability to penetrate deeper into the tubules [68].

It is important to acknowledge the limitations of the current study. Only single moderately curved canals were evaluated, rather than narrow severely curved canals, therefore, further studies should focus on canals with apical curvatures to validate these findings. Rotary files with a convex triangular cross-section were utilized in this study, and the use of Ni-Ti files with alternative cross-sectional designs may influence the outcomes, as variations in cross-sectional geometry alter the contact points between the fractured file fragment and the dentinal wall, thereby affecting the available space around the fragment, which is critical for the apical progression of the sealer and its penetration into dentinal tubules. Although ultrasonic activation was applied consistently across all groups to promote uniform sealer distribution, variation in the initial sealer delivery methods may have influenced the sealer penetration outcomes. Additionally, while the study aimed to simulate clinical conditions, the exact volume of sealer placed in each canal was not precisely quantified, which may also influence penetration depth. Future studies using more controlled application volumes and standardized delivery techniques across all groups are recommended. Cold lateral compaction technique was employed for obturation in this study; however, future investigations should explore alternative obturation techniques with nanoparticles-modified sealers, as previous studies have indicated that both the type of apically separated instrument and the obturation method could significantly impact the sealing ability [18, 60–62, 69, 70]. Although SEM provides high-resolution imaging, it is limited to a single plane and cannot fully capture the curved, three-dimensional wavy course of dentinal tubules. Moreover, transverse sectioning at the junction between the separated file and the obturation material proved technically challenging and inconsistent. Further studies are recommended to use CLSM with transverse sections, which may provide better visualization of sealer penetration and may help overcome these limitations. Finally, the extrapolation of the present findings to in vivo conditions should be done with caution, as tubular sealer penetration is only one of several factors that can influence treatment outcomes following file separation. Additionally, it is crucial to highlight the importance of sealer adaptation to the dentinal canal walls and the prevention of gap formation to gain a more comprehensive understanding of the factors contributing to the success of root canal treatment.

In conclusion, within the limitations of this in vitro study, conventional bioceramic sealer and resin-based sealer modified with silver nanoparticles exhibit more tubular penetration depth when ultrasonically activated over apically separated file in moderately curved canals compared to conventional resin-based sealer and bioceramic sealer modified with bioactive glass nanoparticles.

## Data Availability

The datasets generated and analysed during the current study are available from the corresponding author on reasonable request.

## Abbreviations

AgNPs: Silver nanoparticles
BC: Bioceramic sealer
BC/BG: Bioceramic sealer modified with bioactive glass nanoparticles powder
BGNPs: Bioactive glass nanoparticles
EDTA: Ethylenediaminetetraacetic acid
FTIR: Fourier transform infra-red spectroscopy
NaOCl: Sodium hypochlorite
Rs: Resin-based sealer
Rs/Ag: Resin-based sealer modified with silver nanoparticles powder
SEM: Scanning electron microscope
TEM: Transmission electron microscopy
XRD: X-ray diffraction

## References

[1] Balguerie E, van der Sluis L, Vallaeys K, Gurgel-Georgelin M, Diemer F. Sealer penetration and adaptation in the dentinal tubules: a scanning electron microscopic study. J Endod. 2011;37(11):1576–9. 22000467 10.1016/j.joen.2011.07.005

[2] Madarati AA, Hunter MJ, Dummer PM. Management of intracanal separated instruments. J Endod. 2013;39(5):569–81. 23611371 10.1016/j.joen.2012.12.033

[3] McGuigan M, Louca C, Duncan H. Clinical decision-making after endodontic instrument fracture. Br Dent J. 2013;214(8):395–400. 23619858 10.1038/sj.bdj.2013.379

[4] Spili P, Parashos P, Messer HH. The impact of instrument fracture on outcome of endodontic treatment. J Endod. 2005;31(12):845–50. 16306815 10.1097/01.don.0000164127.62864.7c

[5] Panitvisai P, Parunnit P, Sathorn C, Messer HH. Impact of a retained instrument on treatment outcome: a systematic review and meta-analysis. J Endod. 2010;36(5):775–80. 20416418 10.1016/j.joen.2009.12.029

[6] Caceres C, Larrain MR, Monsalve M, Bengoa FP. Dentinal tubule penetration and adaptation of bio-C sealer and AH-plus: A comparative SEM evaluation. Eur Endod J. 2021;6(2):216. 34047295 10.14744/eej.2020.96658 PMC8461482

[7] El Hachem R, Khalil I, Le Brun G, Pellen F, Le Jeune B, Daou M, El Osta N, Naaman A, Abboud M. Dentinal tubule penetration of AH plus, BC sealer and a novel tricalcium silicate sealer: a confocal laser scanning microscopy study. Clin Oral Investig. 2019;23:1871–6. 30225679 10.1007/s00784-018-2632-6

[8] Omaia M, Sabry H, Shaker M. Evaluation of dentinal tubules penetration of bio-ceramic and resin root Canal sealers using different obturation techniques: an in-vitro study. Adv Dent J. 2023;5(2):449–61.

[9] Jeevanandam J, Barhoum A, Chan YS, Dufresne A, Danquah MK. Review on nanoparticles and nanostructured materials: history, sources, toxicity and regulations. Beilstein J Nanotechnol. 2018;9(1):1050–74. 29719757 10.3762/bjnano.9.98 PMC5905289

[10] Khan I, Saeed K, Khan I. Nanoparticles: properties, applications and toxicities. Arab J Chem. 2019;12(7):908–31.

[11] Barros J, Silva M, Rodrigues M, Alves F, Lopes M, Pina-Vaz I, Siqueira J Jr. Antibacterial, physicochemical and mechanical properties of endodontic sealers containing quaternary ammonium polyethylenimine nanoparticles. Int Endod J. 2014;47(8):725–34. 24134748 10.1111/iej.12207

[12] ElKateb WM, Massoud AG, Mokhless NA, Shalaby TI. Measurement of tubular penetration depth of three types of nanopartcles mixed with endodontic sealer using scanning Electron microscope (An in vitro Study). Am J Sci. 2015;11(11):111–22.

[13] Jung M-K, Park S-C, Kim Y-J, Park J-T, Knowles JC, Park J-H, Dashnyam K, Jun S-K, Lee H-H, Lee J-H. Premixed calcium silicate-based root Canal sealer reinforced with bioactive glass nanoparticles to improve biological properties. Pharmaceutics. 2022;14(9):1903. 36145651 10.3390/pharmaceutics14091903 PMC9506183

[14] Guimarães BM, Amoroso-Silva PA, Alcalde MP, Marciano MA, de Andrade FB, Duarte MAH. Influence of ultrasonic activation of 4 root Canal sealers on the filling quality. J Endod. 2014;40(7):964–8. 24935544 10.1016/j.joen.2013.11.016

[15] Faul F, Erdfelder E, Lang A-G, Buchner A. G* power 3: A flexible statistical power analysis program for the social, behavioral, and biomedical sciences. Behav Res Methods. 2007;39(2):175–91. 17695343 10.3758/bf03193146

[16] Schneider SW. A comparison of Canal preparations in straight and curved root Canals. Oral Surg Oral Med Oral Pathol. 1971;32(2):271–5. 5284110 10.1016/0030-4220(71)90230-1

[17] Chen H, Zhao X, Qiu Y, Xu D, Cui L, Wu B. The tubular penetration depth and adaption of four sealers: a scanning electron microscopic study. Biomed Res Int. 2017;2017(1):2946524. 29479539 10.1155/2017/2946524 PMC5804396

[18] Eymirli A, Uzunoğlu Özyürek E, Serper A. Sealer penetration: effect of separated file’s cross-section, taper and motion characteristics. Clin Oral Investig. 2021;25:1077–84. 32562075 10.1007/s00784-020-03404-3

[19] Wang H, Qiao X, Chen J, Ding S. Preparation of silver nanoparticles by chemical reduction method. Colloids Surf Physicochem Eng Asp. 2005;256(2–3):111–5.

[20] Brinker CJ, Scherer GW. Sol-gel science: the physics and chemistry of sol-gel processing. Academic; 2013.

[21] Hench LL, West JK. The sol-gel process. Chem Rev. 1990;90(1):33–72.

[22] Seung J, Weir MD, Melo MAS, Romberg E, Nosrat A, Xu HHK, Tordik PA. A modified resin sealer: physical and antibacterial properties. J Endod. 2018;44(10):1553–7. 30174102 10.1016/j.joen.2018.06.016

[23] Viapiana R, Guerreiro-Tanomaru JM, Hungaro-Duarte MA, Tanomaru-Filho M, Camilleri J. Chemical characterization and bioactivity of epoxy resin and Portland cement-based sealers with Niobium and zirconium oxide radiopacifiers. Dent Mater. 2014;30(9):1005–20. 24950807 10.1016/j.dental.2014.05.007

[24] Ltd SE. Simple randomisation service. 2022.

[25] De Bem IA, de Oliveira RA, Weissheimer T, Bier CAS, Só MVR, da Rosa RA. Effect of ultrasonic activation of endodontic sealers on intratubular penetration and bond strength to root dentin. J Endod. 2020;46(9):1302–8. 32615175 10.1016/j.joen.2020.06.014

[26] Rueden CT, Schindelin J, Hiner MC, DeZonia BE, Walter AE, Arena ET, Eliceiri KW. ImageJ2: ImageJ for the next generation of scientific image data. BMC Bioinformatics. 2017;18:1–26. 29187165 10.1186/s12859-017-1934-z PMC5708080

[27] Alrehily FA. Assessing the inter-observer and intra-observer reliability of radiographic measurements for size-specific dose estimates. BMC Med Imaging. 2024;24(1):209. 39134971 10.1186/s12880-024-01397-z PMC11318122

[28] Anandalakshmi K, Venugobal J, Ramasamy V. Characterization of silver nanoparticles by green synthesis method using pedalium murex leaf extract and their antibacterial activity. Appl Nanosci. 2016;6:399–408.

[29] Abu Zeid ST, Edrees HY. Hydration characterization of two generations of MTA-based root Canal sealers. Appl Sci. 2022;12(7):3517.

[30] Ghazizadeh A, Haddadi SA, Mahdavian M. The effect of sol–gel surface modified silver nanoparticles on the protective properties of the epoxy coating. RSC Adv. 2016;6(23):18996–9006.

[31] Belhachem A, Douahi O, Yahia Y, Cherifi Z, Amiar A, Boudia F, Meghabar R, Toumi H. Study of the antibacterial activity of hybrid nanocomposites metal oxide/alginate synthesized for therapeutic purposes. Discov Med. 2024;1(1):1–19.

[32] Lin GSS, Sim DHH, Luddin N, Lai JCH, Abd Ghani H, Noorani TY. Fabrication and characterisation of novel algin incorporated bioactive-glass 58S calcium-silicate-based root Canal sealer. J Dent Sci. 2023;18(2):604–12. 37021270 10.1016/j.jds.2022.08.012 PMC10068582

[33] Leenakul W, Tunkasiri T, Tongsiri N, Pengpat K, Ruangsuriya J. Effect of sintering temperature variations on fabrication of 45S5 bioactive glass-ceramics using rice husk as a source for silica. Mater Sci Eng C. 2016;61:695–704. 10.1016/j.msec.2015.12.029 26838899

[34] Nabian N, Jahanshahi M, Rabiee SM. Synthesis of nano-bioactive glass–ceramic powders and its in vitro bioactivity study in bovine serum albumin protein. J Mol Struct. 2011;998(1–3):37–41.

[35] Siqueira JF Jr. Aetiology of root Canal treatment failure: why well-treated teeth can fail. Int Endod J. 2001;34(1):1–10. 11307374 10.1046/j.1365-2591.2001.00396.x

[36] Gomes MS, Vieira RM, Böttcher DE, Plotino G, Celeste RK, Rossi-Fedele G. Clinical fracture incidence of rotary and reciprocating NiTi files: A systematic review and meta‐regression. Aust Endod J. 2021;47(2):372–85. 33410578 10.1111/aej.12484

[37] McGuigan M, Louca C, Duncan H. Endodontic instrument fracture: causes and prevention. Br Dent J. 2013;214(7):341–8. 23579132 10.1038/sj.bdj.2013.324

[38] Madarati A, Watts D, Qualtrough A. Factors contributing to the separation of endodontic files. Br Dent J. 2008;204(5):241–5. 18327187 10.1038/bdj.2008.152

[39] Şen B, Pişkin B, Baran N. The effect of tubular penetration of root Canal sealers on dye microleakage. Int Endod J. 1996;29(1):23–8. 9206408 10.1111/j.1365-2591.1996.tb01355.x

[40] Marissa C, Usman M, Suprastiwi E, Erdiani A, Meidyawati R. Comparison of dentinal tubular penetration of three bioceramic sealers. Int J Appl Pharm. 2020;12:23–6.

[41] Ricucci D, Siqueira JF Jr, Rôças IN. Pulp response to periodontal disease: novel observations help clarify the processes of tissue breakdown and infection. J Endod. 2021;47(5):740–54. 33610600 10.1016/j.joen.2021.02.005

[42] Lang NP, Lindhe J. Clinical periodontology and implant dentistry. 2 ed. Volume Set: Wiley; 2015.

[43] Rahimi M, Jainaen A, Parashos P, Messer HH. Bonding of resin-based sealers to root dentin. J Endod. 2009;35(1):121–4. 19084140 10.1016/j.joen.2008.10.009

[44] Versiani M, Carvalho-Junior J, Padilha M, Lacey S, Pascon E, Sousa‐Neto M. A comparative study of physicochemical properties of AH PlusTM and epiphanytm root Canal sealants. Int Endod J. 2006;39(6):464–71. 16674741 10.1111/j.1365-2591.2006.01105.x

[45] Collado-González M, García‐Bernal D, Oñate‐Sánchez R, Ortolani‐Seltenerich P, Lozano A, Forner L, Llena C, Rodríguez‐Lozano F. Biocompatibility of three new calcium silicate‐based endodontic sealers on human periodontal ligament stem cells. Int Endod J. 2017;50(9):875–84. 27666949 10.1111/iej.12703

[46] Bukhari S, Karabucak B. The antimicrobial effect of bioceramic sealer on an 8-week matured Enterococcus faecalis biofilm attached to root Canal dentinal surface. J Endod. 2019;45(8):1047–52. 31160079 10.1016/j.joen.2019.04.004

[47] Camps J, Jeanneau C, El Ayachi I, Laurent P, About I. Bioactivity of a calcium silicate–based endodontic cement (BioRoot RCS): interactions with human periodontal ligament cells in vitro. J Endod. 2015;41(9):1469–73. 26001857 10.1016/j.joen.2015.04.011

[48] Ginebra M-P, Fernandez E, De Maeyer E, Verbeeck R, Boltong M, Ginebra J, Driessens F, Planell J. Setting reaction and hardening of an apatitic calcium phosphate cement. J Dent Res. 1997;76(4):905–12. 9126187 10.1177/00220345970760041201

[49] Afkhami F, Nasri S, Valizadeh S. Evaluation of the antibacterial effect of AH plus sealer modified with silver nanoparticles. 2021. 10.1186/s12903-021-01924-2 PMC8588647 34772398

[50] Afkhami F, Nasri S, Valizadeh S. Bacterial leakage assessment in root canals sealed with AH plus sealer modified with silver nanoparticles. BMC Oral Health. 2021;21(1):577. 34772398 10.1186/s12903-021-01924-2 PMC8588647

[51] Teixeira ABV, Vidal CL, Albiasetti T, de Castro DT, Dos Reis AC. Influence of adding nanoparticles of silver vanadate on antibacterial effect and physicochemical properties of endodontic sealers. Iran Endod J. 2019;14(1):7. 36879594 10.22037/iej.v14i1.22519 PMC9984810

[52] Baras BH, Melo MAS, Sun J, Oates TW, Weir MD, Xie X, Bai Y, Xu HH. Novel endodontic sealer with dual strategies of dimethylaminohexadecyl methacrylate and nanoparticles of silver to inhibit root Canal biofilms. Dent Mater. 2019;35(8):1117–29. 31128937 10.1016/j.dental.2019.05.014

[53] Wiesse P, Silva-Sousa Y, Pereira R, Estrela C, Domingues L, Pécora JD, Sousa‐Neto MD. Effect of ultrasonic and Sonic activation of root Canal sealers on the push‐out bond strength and interfacial adaptation to root Canal dentine. Int Endod J. 2018;51(1):102–11. 28543092 10.1111/iej.12794

[54] Bittmann B, Haupert F, Schlarb AK. Ultrasonic dispersion of inorganic nanoparticles in epoxy resin. Ultrason Sonochem. 2009;16(5):622–8. 19231271 10.1016/j.ultsonch.2009.01.006

[55] Dasari L, Anwarullah A, Mandava J, Konagala RK, Karumuri S, Chellapilla PK. Influence of obturation technique on penetration depth and adaptation of a bioceramic root Canal sealer. J Conserv Dent Endod. 2020;23(5):505–11. 10.4103/JCD.JCD_450_20 PMC8066675 33911361

[56] Ferreira EHRG, Rosas CAP, Limoeiro AGS, Lima SN, Silva RA, Seckler INB, De Martin AS, Nascimento WM, Bueno CES. Effect of obturation technique on dentinal tubule penetration of two tricalcium silicate–based sealers: ex vivo study. Aust Endod J. 2024;50(1):148–56. 38146083 10.1111/aej.12825

[57] Peng L, Ye L, Tan H, Zhou X. Outcome of root Canal obturation by warm gutta-percha versus cold lateral condensation: a meta-analysis. J Endod. 2007;33(2):106–9. 17258624 10.1016/j.joen.2006.09.010

[58] Ho ESS, Chang JWW, Cheung GSP. Quality of root Canal fillings using three gutta-percha obturation techniques. Restor Dent Endod. 2016;41(1):22–8. 26877987 10.5395/rde.2016.41.1.22 PMC4751203

[59] Mancino D, Kharouf N, Cabiddu M, Bukiet F, Haïkel Y. Microscopic and chemical evaluation of the filling quality of five obturation techniques in oval-shaped root canals. Clin Oral Investig. 2021;25:3757–65. 33244706 10.1007/s00784-020-03703-9

[60] Aktemur Türker S, Uzunoğlu Özyürek E, Tek V. The effect of different obturation methods on sealer penetration alongside apically separated rotary nickel–titanium instruments: A confocal laser scanning microscopy study. Microsc Res Tech. 2020;83(6):720–6. 32159907 10.1002/jemt.23461

[61] Altundasar E, Sahin C, Ozcelik B, Cehreli ZC. Sealing properties of different obturation systems applied over apically fractured rotary nickel–titanium files. J Endod. 2008;34(2):194–7. 18215680 10.1016/j.joen.2007.10.023

[62] Mehrotra A, Gutte NH, Mishra R, Ughade SP, Nanditha S. Sealing ability of different obturating techniques in apically separated rotary files: an: in vitro: study. J Pharm Bioallied Sci. 2022;14(Suppl 1):S884–7. 36110826 10.4103/jpbs.jpbs_911_21 PMC9469406

[63] Lee JK, Kwak SW, Ha J-H, Lee W, Kim H-C. Physicochemical properties of epoxy resin-based and bioceramic‐based root Canal sealers. Bioinorg Chem Appl. 2017;2017(1):2582849. 28210204 10.1155/2017/2582849 PMC5292198

[64] Kishen A, Shi Z, Shrestha A, Neoh KG. An investigation on the antibacterial and antibiofilm efficacy of cationic nanoparticulates for root Canal disinfection. J Endod. 2008;34(12):1515–20. 19026885 10.1016/j.joen.2008.08.035

[65] Kaplan AE, Ormaechea M, Picca M, Canzobre M, Ubios A. Rheological properties and biocompatibility of endodontic sealers. Int Endod J. 2003;36(8):527–32. 12887381 10.1046/j.1365-2591.2003.00683.x

[66] Wang Y, Liu S, Dong Y. In vitro study of dentinal tubule penetration and filling quality of bioceramic sealer. PLoS ONE. 2018;13(2):e0192248. 29390037 10.1371/journal.pone.0192248 PMC5794174

[67] Al-Haddad A, Che Ab Aziz ZA. Bioceramic-based root Canal sealers: a review. Int J Biomater. 2016;2016(1):9753210. 27242904 10.1155/2016/9753210 PMC4868912

[68] Roberts HW, Toth JM, Berzins DW, Charlton DG. Mineral trioxide aggregate material use in endodontic treatment: a review of the literature. Dent Mater. 2008;24(2):149–64. 17586038 10.1016/j.dental.2007.04.007

[69] Türker SA, Uzunoğlu-Özyürek E, Kaşikçi S, Öndeş M, Geneci F, Çelik HH. Filling quality of several obturation techniques in the presence of apically separated instruments: A Micro‐CT study. Microsc Res Tech. 2021;84(6):1265–71. 33378798 10.1002/jemt.23685

[70] Sevinc AT, Emel UO, Vildan T. The effect of different obturation methods on sealer penetration alongside apically separated rotary nickel-titanium instruments: A confocal laser scanning microscopy study. 10.1002/jemt.23461 32159907

